# Effects of season and water quality on community structure of planktonic eukaryotes in the Chaohu Lake Basin

**DOI:** 10.3389/fmicb.2024.1424277

**Published:** 2024-08-14

**Authors:** Yan Zhang, Maozhen Han, Li Wu, Guoao Ding, Kai Liu, Kui He, Jingqiu Zhao, Yiwen Liao, Yun Gao, Cui Zhang

**Affiliations:** ^1^School of Biological and Food Engineering, Hefei Normal University, Hefei, China; ^2^School of Life Sciences, Anhui Medical University, Hefei, China; ^3^Freshwater Fisheries Research Center, Chinese Academy of Fishery Sciences, Wuxi, China; ^4^Scientific Observing and Experimental Station of Fishery Resources and Environment in the Lower Reaches of the Changjiang River, Ministry of Agriculture and Rural Affairs, Wuxi, China

**Keywords:** aquatic ecosystem, aquatic physicochemical parameters, Chaohu Lake Basin, environmental DNA, eukaryotic plankton community

## Abstract

**Introduction:**

Analyzing the correlation between planktonic eukaryotic communities (PECs) and aquatic physicochemical parameters (APPs) provides important references for predicting the impact of climate change and human activities on aquatic ecosystems.

**Methods:**

To assess the influence of seasons and APPs on PEC structures in lakes and rivers, we utilized high-throughput sequencing of the 18S rRNA gene to analyze PEC structures in a lake and seven rivers in the Chaohu Lake Basin and analyzed their correlations with APPs.

**Results:**

Our results revealed that PEC structure was significantly affected by season, with the highest α-diversity observed in summer. Furthermore, we identified several APPs, including water temperature, conductivity, dissolved oxygen, pH, phosphate, total phosphorus, trophic level index (TLI), nitrate, ammonia nitrogen, and total nitrogen, that significantly influenced PEC structures. Specifically, we found that *Stephanodiscus hantzschii*, *Simocephalus serrulatus*, *Cryptomonas* sp. CCAC_0109, *Pedospumella encystans*, *Actinochloris sphaerica*, *Chlamydomonas angulosa*, *Gonyostomum semen*, *Skeletonema potamos*, *Chlamydomonas klinobasis*, *Pedospumella* sp., and *Neochlorosarcina negevensis* were significantly correlated to TLI, while *Limnoithona tetraspina*, *Theileria* sp., and *Pseudophyllomitus vesiculosus* were significantly correlated to the water quality index (WQI). However, our random forest regression analysis using the top 100 species was unable to accurately predict the WQI and TLI.

**Discussion:**

These results provide valuable data for evaluating the impact of APPs on PEC and for protecting water resource in the Chaohu Lake Basin.

## Introduction

The health and maintenance of aquatic ecosystems are important ecological issues (Zhang C. et al., 2023; Zhang Z. et al., 2023). Planktonic eukaryotes (PEs) are highly diverse and play crucial roles as producers, consumers, and trophic links in aquatic food webs (Li et al., 2023). They are essential to biogeochemical processes in these ecosystems (Li et al., 2023). Phytoplankton are particularly important in fixing CO_2_ and other elements, converting them into organic matters. Additionally, zooplankton feed on phytoplankton, transferring biogenic elements to higher trophic levels (HTLs) (Li et al., 2023). Therefore, PEs are used as indicators of environmental conditions, reflecting the ecosystem status and living components, and providing information about the abundance of predators, such as fish (Lomartire et al., 2021).

However, PEs are influenced by both climate change and human activities (Ting et al., 2021), which alter the aquatic physicochemical parameters (APPs). For instance, the diversity of PEs has been observed to decline due to excessive disturbance from urban and agricultural activities in the midstream of the Xiaoqing River (Xu et al., 2020). In addition, Zhang C. et al. (2023) and Zhang Z. et al. (2023) found that potential anthropogenic factors such as nutrients and heavy metals strongly influence the spatial co-occurrence patterns and network topology of plankton along the Chinese coastline. Furthermore, PEs that play crucial roles in material cycles are greatly affected by nutrient levels in the aquatic ecosystem (Liu et al., 2022). As a result, water samples from rivers and lakes in urban areas are often used to assess the impacts of anthropogenic activities on the environment (Cai et al., 2018; Jin et al., 2018).

The Chaohu Lake (CL) Basin, with a drainage area of 1.35 × 10^5^ km^2^, is located downstream of the Yangtze River. This region experiences a subtropical humid monsoon climate, characterized by four distinct seasons (Wu et al., 2021). To assess the impact of seasons and human activities on the planktonic eukaryotic community (PEC) structure in lakes and rivers, we conducted a study using high-throughput sequencing of the 18S rRNA gene to measure the PEC structure in different sections of seven rivers and the lake in the CL Basin. We also analyzed their correlations with APPs.

## Materials and methods

### Sample collection and determination of APPs

**Figure 1:**
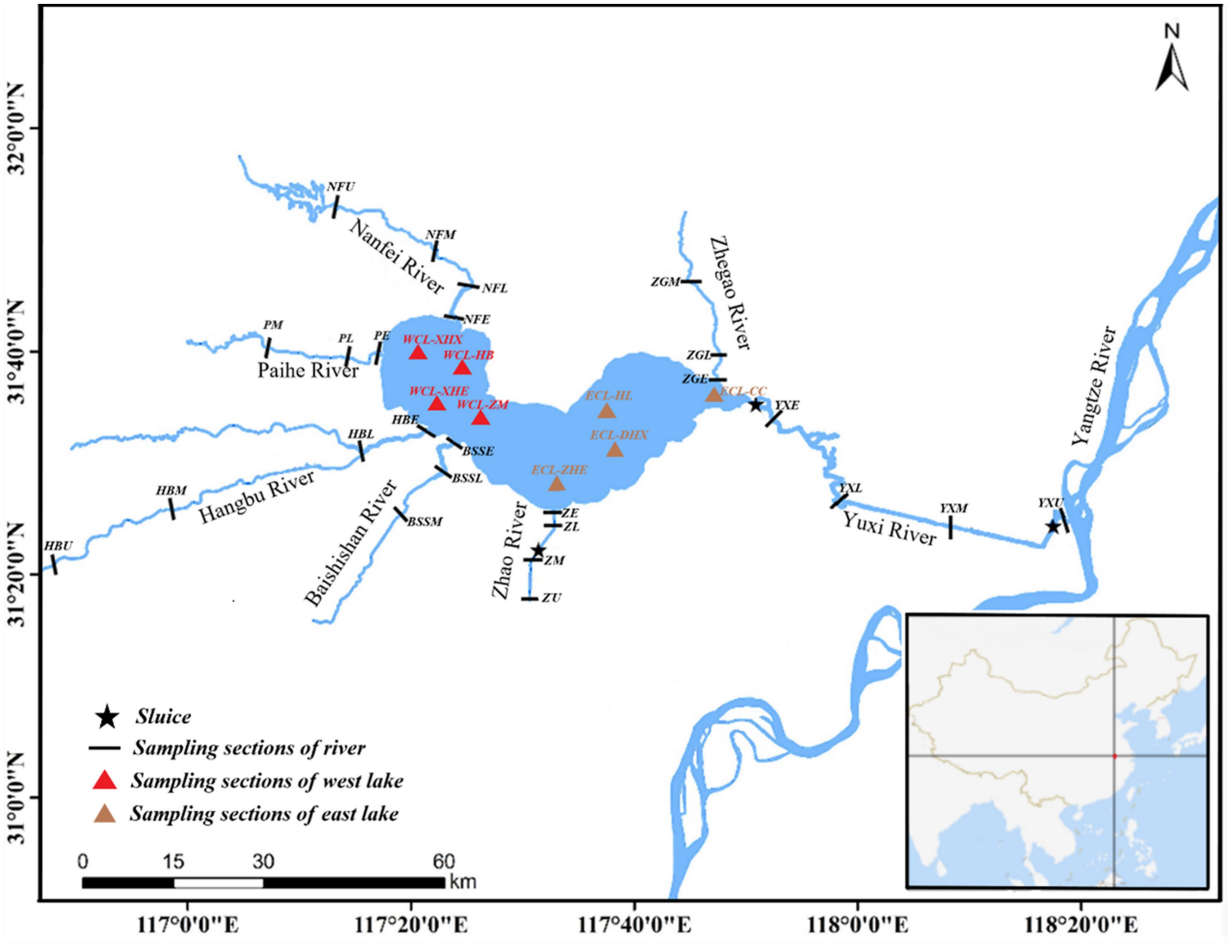
Distribution of sampling sites. WCL, West Chaohu Lake; ECL, East Chaohu Lake; NFU, upstream of the Nanfei River; NFM, midstream of the Nanfei River; NFL, downstream of the Nanfei River; NFE, estuary areas of the Nanfei River; ZGM, midstream of the Zhegao River; ZGL, downstream of the Zhegao River; ZGE, estuary areas of the Zhegao River; YXU, upstream of the Yuxi River; YXM, midstream of the Yuxi River; YXL, downstream of the Yuxi River; YXE, estuary areas of the Yuxi River; ZHU, upstream of the Zhao River; ZHM, midstream of the Zhao River; ZHL, downstream of the Zhao River; ZHE, estuary areas of the Zhao River; BSSM, midstream of the Baishishan River; BSSL, downstream of the Baishishan River; BSSE, estuary areas of the Baishishan River; HBU, upstream of the Hangbu River; HBM, midstream of the Hangbu River; HBL, downstream of the Hangbu River; HBE, estuary areas of the Hangbu River.

Thirty-three sampling sites were located in the West CL (WCL), and East CL (ECL), and different sections of the Nanfei River (NFR), Hangbu River (HBR), Zhao River (ZR), Yuxi River (YXR), Pai River (PR), Baishishan River (BSSR), and Zhegao River (ZGR) as previously described (Wu et al., 2022). Water samples were collected on October 14, 2019 (autumn), January 3 (winter), April 24 (spring), and July 10, 2020 (summer) as previously described (Wu et al., 2022) (see Figure 1).

The physicochemical parameters [water temperature (WT), pH, dissolved oxygen (DO), conductivity, total nitrogen (TN), NH_4_-N, NO_3_-N, NO_2_-N, total phosphorus (TP), PO_4_-P, permanganate index (COD_Mn_), biochemical oxygen demand after 5 days (BOD_5_), and chlorophyll a (Chla)] of water were measured as previously described (Wu et al., 2022). The water quality index (WQI) is a commonly used tool for assessing water quality (Uddin et al., 2021). It combines physical, chemical, and biological factors into a single value ranging from 0 to 100 (Chidiac et al., 2023). However, the trophic level index (TLI) is a more specific indicator for measuring and understanding lake eutrophication (Hu et al., 2024). It ranks lakes based on their productivity and nutrient levels, allowing for the evaluation of water trophic state in response to factors such as nutrient loading (Parparov et al., 2010). WQI and TLI were calculated according to previously described (Liu et al., 2020; Wu et al., 2023).

### Analysis of PEC structure

Water microbiota DNA was extracted according to previously described (Wu et al., 2023). The V9 region of the eukaryotic 18S rRNA gene was amplified using the primer set 1380F and 1510R (Amaral-Zettler et al., 2009). Triplicate PCR products were sequenced on an Illumina Xten platform (Illumina, United States), as previously reported (Liu et al., 2021).

### Data analysis

Principal co-ordinates analysis (PCoA) was performed to analyze the difference of microbiota compositions among seasons using the R ggbiplot package. A boxplot was constructed to show the difference of microbiota α-diversity indices (operational taxonomic units (OTUs), Shannon, Simpson, Chao1, and Ace indices) using the R ggpubr package. Spearman’s correlation analysis between water environmental factors and microbiota compositions was conducted using the R psych, reshape2, and corrplot packages. Distance-based redundancy analysis (db-RDA) of water microbiota compositions and environmental factors was conducted using the R vegan package. Co-occurrence network analysis between microbiota compositions and environmental factors was conducted using the R igraph, psych, and Hmisc packages, and visualized using Gephi 0.9.2. SourceTricker analysis of microbiota compositions was conducted using the SourceTricker script (Knights et al., 2011). Random forest regression of WQI and TLI was conducted using the R randomForest package. Statistical significance was set at *p* < 0.05.

## Results

The APPs exhibited significant seasonal fluctuations (Supplementary Figure S1). WT was significant differences between seasons (*p* < 0.05; Supplementary Figure S1A). The water DO in spring and winter was significantly higher than those in summer and autumn (*p* < 0.05; Supplementary Figure S1B). The transparency in summer was significantly lower than that in winter (*p* < 0.05; Supplementary Figure S1C). The water pH in spring and autumn was significantly higher than that in summer and winter (*p* < 0.05; Supplementary Figure S1D). There were no significant seasonal differences in BOD_5_ or COD_Mn_ (*p* < 0.05; Supplementary Figures S1E,F). Water conductivity in summer was significantly lower than those in the other seasons (*p* < 0.05; Supplementary Figure S1G). The water TN in spring was significantly lower than those in summer and winter (*p* < 0.05; Supplementary Figure S1H). The water NH_4_-N concentration in winter was significantly higher than that in spring and summer (*p* < 0.05; Supplementary Figure S1I), whereas the water NO_2_-N concentration in winter was significantly lower than that in spring and summer (*p* < 0.05; Supplementary Figure S1J). The water NO_3_-N concentrations in spring and autumn were significantly lower than those in summer and winter (*p* < 0.05; Supplementary Figure S1K). The water TP in summer was significantly higher than those in spring and winter (*p* < 0.05; Supplementary Figure S1L), and the water PO_4_-P concentration in winter was significantly lower than those in other seasons (*p* < 0.05; Supplementary Figure S1M). The water chlorophyll-a (chl-a) concentration in spring was significantly lower than those in the other seasons, whereas the chl-a concentration in autumn was significantly higher than those in the other seasons (*p* < 0.05; Supplementary Figure S1N). The TLIs in spring and winter were significantly lower than those in summer and autumn (*p* < 0.05; Supplementary Figure S1O), whereas the WQI in summer was significantly lower than that in spring (*p* < 0.05; Supplementary Figure S1P). For rivers and lakes, the water DOs of NFH, PH, and ZGH were significantly lower than those of ECH and WCH, and the transparency of HBH was significantly higher than those of the other rivers and the lake, whereas the transparencies of ECH and WCH were significantly lower than those of HBH, SBBH, ZH, YXH, and ZGH (*p* < 0.05; Supplementary Figure S2). The water pH was significantly higher than that of the rivers. The water BOD_5_ of NFH and PH was significantly higher than that of CL (*p* < 0.05; Supplementary Figure S2). The concentrations of nitrogen and phosphorous in NFH and PH were significantly higher than those in other rivers and CL, indicating that the water quality of these two rivers was at a higher risk of eutrophication than that of the other rivers. The WQI also indicated that the water quality of these two rivers was the worst, but the TLI indicated that in addition to these two rivers, the TLI of WCH was also significantly higher than that of the other rivers and ECH (*p* < 0.05; Supplementary Figure S2). Furthermore, the significant increases of TN, NH_4_-N, TP, and PO_4_-P contents in the NFR and PR probably was the main reason for the significantly higher contents of these nutrients in the WCL than in the ECL (*p* < 0.05; Supplementary Figure S2). However, although the concentrations of water NO_3_-N and NO_2_-N in NFR and PR were significantly higher than those in other rivers (*p* < 0.05; Supplementary Figure S2), there was no significant difference in water NO_3_-N and NO_2_-N concentrations between WCL and ECL (*p* > 0.05; Supplementary Figure S2).

**Figure 2:**
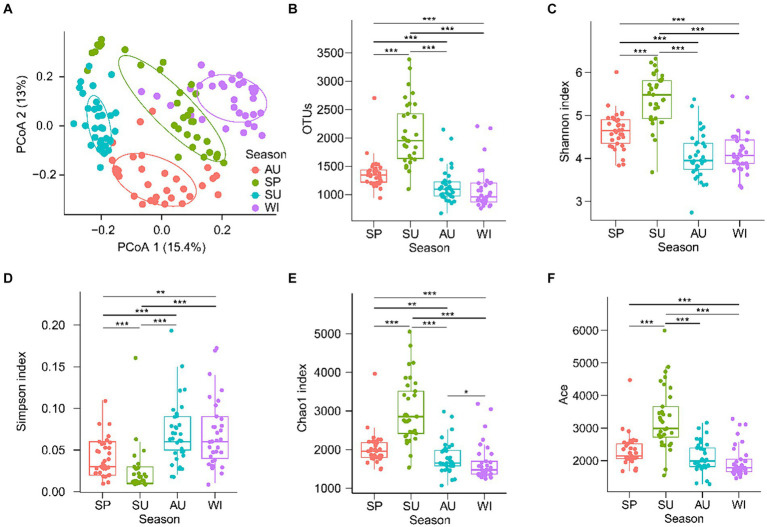
Changes of planktonic eukaryotic community structure and diversity in the Chaohu Lake basin among different seasons. **(A)** Principal co-ordinates analysis (PCoA) profile. **(B)** Operational taxonomic units (OTUs) number. **(C)** Shannon index. **(D)** Simpson index. **(E)** Chao1 index. **(F)** Ace index. SP, spring; SU, summer; AU, autumn; WI, winter. ^**^p < 0.01; ^***^p < 0.001.

Samples obtain different numbers of original reads and high-quality tags through high-throughput sequencing, which interferes subsequent analysis results (Fierer et al., 2012). Therefore, the resample method is usually used to flatten the high-quality tags of all samples to eliminate the influence of sequencing depth on the subsequent analysis results (Yan et al., 2016; Ni et al., 2021). After quality control, 45,009 high-quality 18S rDNA tags from PECs were randomly resampled from each sample for subsequent analysis. PCoA with PERMANOVA showed that PECs were significantly different among the seasons (*F* = 13.321, *p* = 0.005; Figure 2A). The OTU number, Shannon, Chao1, and ACE indices of the summer PECs were significantly higher than those of other seasons, whereas the Simpson index of the summer PECs was significantly lower than that of other seasons (*p* < 0.05; Figures 2B–F). These results indicated that the structure of PECs in the CL Basin was significantly influenced by season, with the highest α-diversity observed in summer.

**Figure 3:**
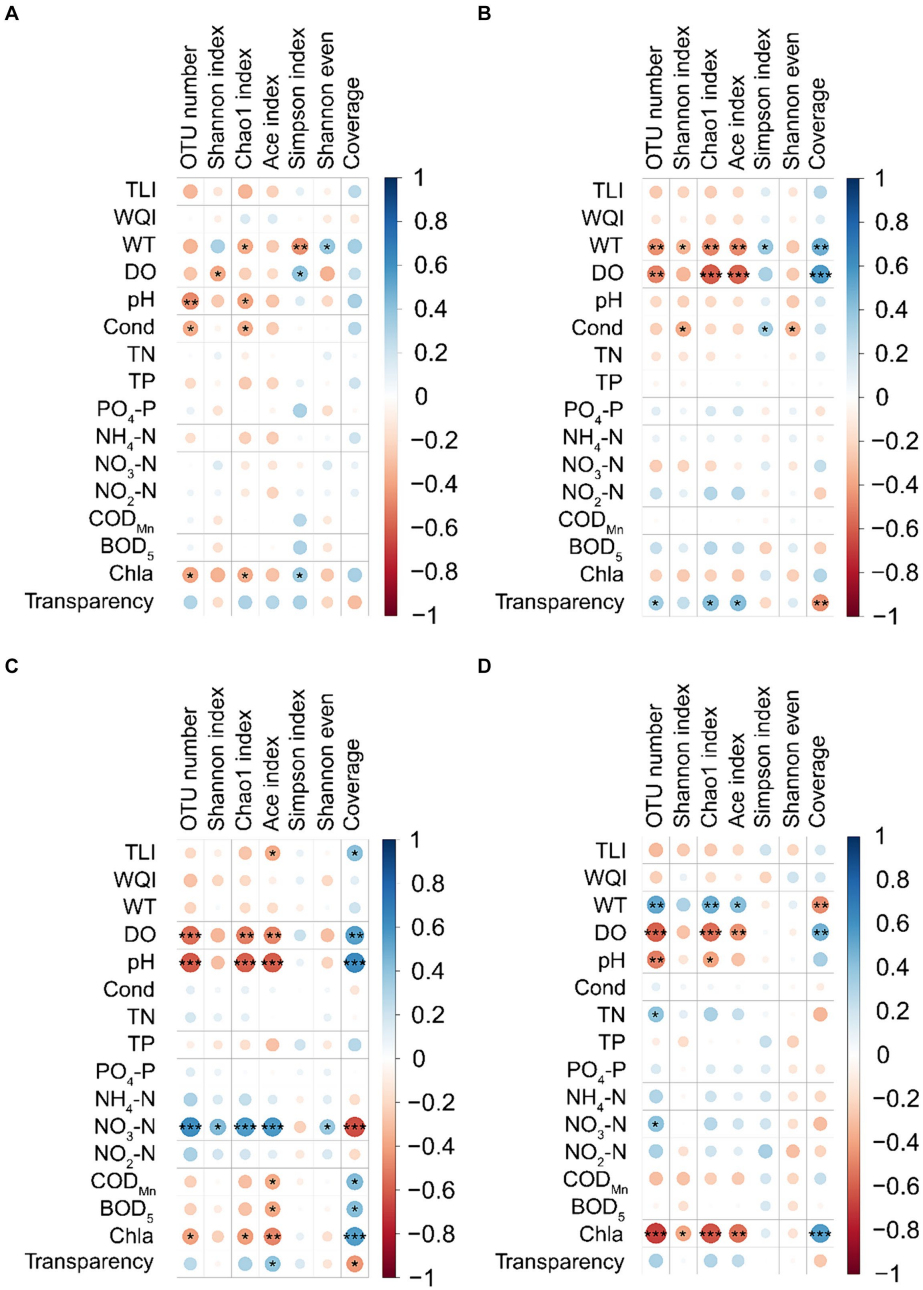
Correlation between water physicochemical parameters and α-diversity indices of planktonic eukaryotic communities in the Chaohu Lake basin. **(A)** Spring. **(B)** Summer. **(C)** Autumn. **(D)** Winter. OTU, operational taxonomic units; TLI, trophic level index; WQI, water quality index; WT, water temperature; DO, dissolved oxygens; Cond, conductivity; TN, total nitrogen; TP, total phosphorus; COD_Mn_, permanganate index; BOD_5_, biochemical oxygen demand after 5 days; Chla, chlorophyll a. ^*^p < 0.05; ^**^p < 0.01; ^***^p < 0.001.

Spearman’s correlation analysis showed that the effect of water physicochemical parameters on the α-diversity indices of PECs varied in different seasons. In spring, WT was significantly positively correlated with the Shannon even index, whereas it was significantly negatively correlated with the Chao1 and Simpson indices. DO was significantly positively correlated with the Simpson index but significantly negatively correlated with the Shannon index. Water pH and conductivity were significantly negatively correlated with OTU number and the Chao1 index. Chl-a was significantly positively correlated with the Simpson index, whereas it was significantly negatively correlated with OTU number and Chao1 index (*p* < 0.05; Figure 3A). In summer, WT was significantly positively correlated with Simpson index and Good’s coverage, whereas it was significantly negatively correlated with OTU number, Shannon, Chao1, and ACE indices. DO was significantly positively correlated with Good’s coverage, but significantly negatively correlated with OTU number, Chao1, and ACE indices. Conductivity was significantly positively correlated with the Simpson index but significantly negatively correlated with the Shannon and Shannon even indices. Water transparency was significantly positively correlated with OTU number, Chao1, and ACE indices, whereas it was significantly negatively correlated with Good’s coverage (*p* < 0.05; Figure 3B). In autumn, TLI was significantly positively correlated with Good’s coverage, but significantly negatively correlated with the ACE index. DO, pH, and chl-a were significantly positively correlated with Good’s coverage, whereas they were significantly negatively correlated with OTU number, Chao1, and ACE indices. Water NO_3_-N was significantly positively correlated with OTU number, Shannon, Chao1, ACE, and Shannon even indices, whereas it was significantly negatively correlated with Good’s coverage. COD_Mn_ and BOD_5_ significantly positively correlated with Good’s coverage, whereas they were significantly negatively correlated with ACE index. Water transparency was significantly positively correlated with ACE index, whereas it was significantly negatively correlated with Good’s coverage (*p* < 0.05; Figure 3C). In winter, WT was significantly positively correlated with OTU number, Chao1 and ACE indices, whereas it was significantly negatively correlated with Good’s coverage. DO was significantly positively correlated with Good’s coverage, but significantly negatively correlated with OTU number, Chao1, and ACE indices. Water pH was significantly negatively correlated with OTU number and the Chao1 index. Water TN and NO_3_-N were significantly positively correlated with OTU number. Water chl-a was significantly positively correlated with Good’s coverage, whereas it was significantly negatively correlated with OTU number, Shannon, Chao1, and ACE indices (*p* < 0.05; Figure 3D). Notably, the WQI was not significantly correlated with the α-diversity indices of PECs in any season.

**Figure 4:**
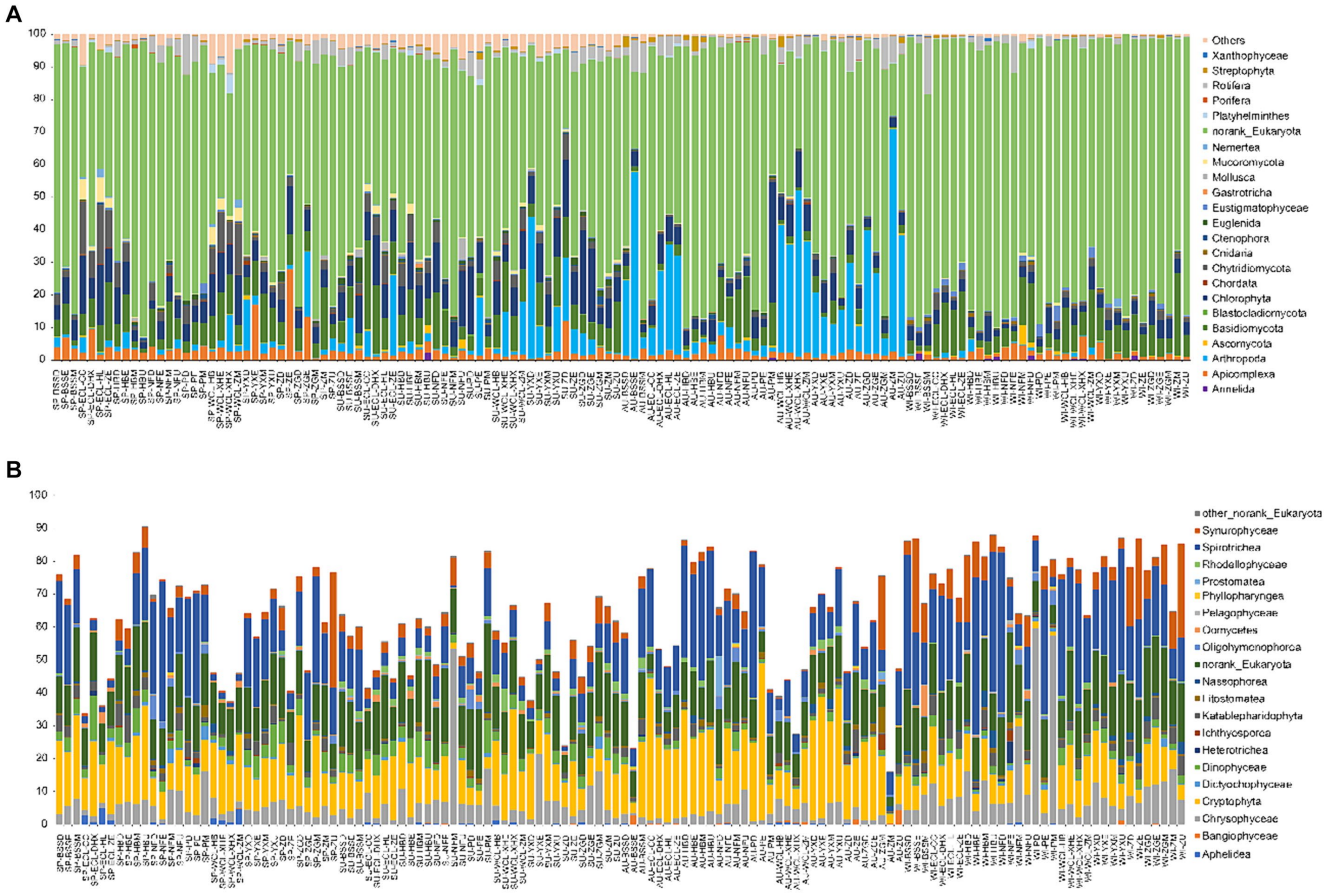
Compositions of dominant phyla **(A)** and classes of norank_Eukaryota **(B)** in planktonic eukaryotic communities in the Chaohu Lake basin. WCL, West Chaohu Lake; ECL, East Chaohu Lake; NFU, upstream of the Nanfei River; NFM, midstream of the Nanfei River; NFL, downstream of the Nanfei River; NFE, estuary areas of the Nanfei River; ZGM, midstream of the Zhegao River; ZGL, downstream of the Zhegao River; ZGE, estuary areas of the Zhegao River; YXU, upstream of the Yuxi River; YXM, midstream of the Yuxi River; YXL, downstream of the Yuxi River; YXE, estuary areas of the Yuxi River; ZHU, upstream of the Zhao River; ZHM, midstream of the Zhao River; ZHL, downstream of the Zhao River; ZHE, estuary areas of the Zhao River; BSSM, midstream of the Baishishan River; BSSL, downstream of the Baishishan River; BSSE, estuary areas of the Baishishan River; HBU, upstream of the Hangbu River; HBM, midstream of the Hangbu River; HBL, downstream of the Hangbu River; HBE, estuary areas of the Hangbu River. SP, spring; SU, summer; AU, autumn; WI, winter.

At the phylum level, most sequences were assigned to norank_Eukaryota. Additionally, Annelida, Apicomplexa, Arthropoda, Bacillariophyta, Basidiomycota, Chlorophyta, Chordata, Cnidaria, Ctenophora, Eustigmatophyceae, Gastrotricha, Mollusca, Mucoromycota, Nemertea, Platyhelminthes, Porifera, Rotifera, Streptophyta, and Xanthophyceae dominated the PECs (Figure 4A). All dominant phyla exhibited significant seasonal differences (Kruskal-Wallis rank sum test, *p* < 0.05; Supplementary Figure S3), especially the relative abundances of Apicomplexa and Nematoda were significantly higher in spring than in the other seasons, whereas the relative abundance of Xanthophyceae was significantly lower in spring than in the other seasons. The relative abundances of Blastocladiomycota, Chlorophyta, Euglenida, and Mollusca were significantly higher in the summer than in the other seasons. The relative abundances of Arthropoda and Streptophyta were significantly higher in autumn than in the other seasons, whereas the relative abundance of Bacillariophyta was significantly lower in autumn than in the other seasons. The relative abundances of Annelida, Basidiomycota, Eustigmatophyceae, and Nemertea were significantly higher in winter than in the other seasons, whereas those of Euglenida, Gastrotricha, Mollusca, Porifera, and Rotifera were significantly lower in winter than in the other seasons (Supplementary Figure S3). Moreover, in the unranked Eukaryota, Spirotrichea, Cryptophyta, Chrysophyceae, Synurophyceae, Dinophyceae, Katablepharidophyta, Litostomatea, Rhodellophyceae, Oligohymenophorea, Oomycetes, Dictyochophyceae, Nassophorea, Aphelidea, Ichthyosporea, Bangiophyceae, Heterotrichea, Prostomatea, Pelagophyceae, Phyllopharyngea, and Colpodea were dominant classes (Figure 4B).

**Figure 5:**
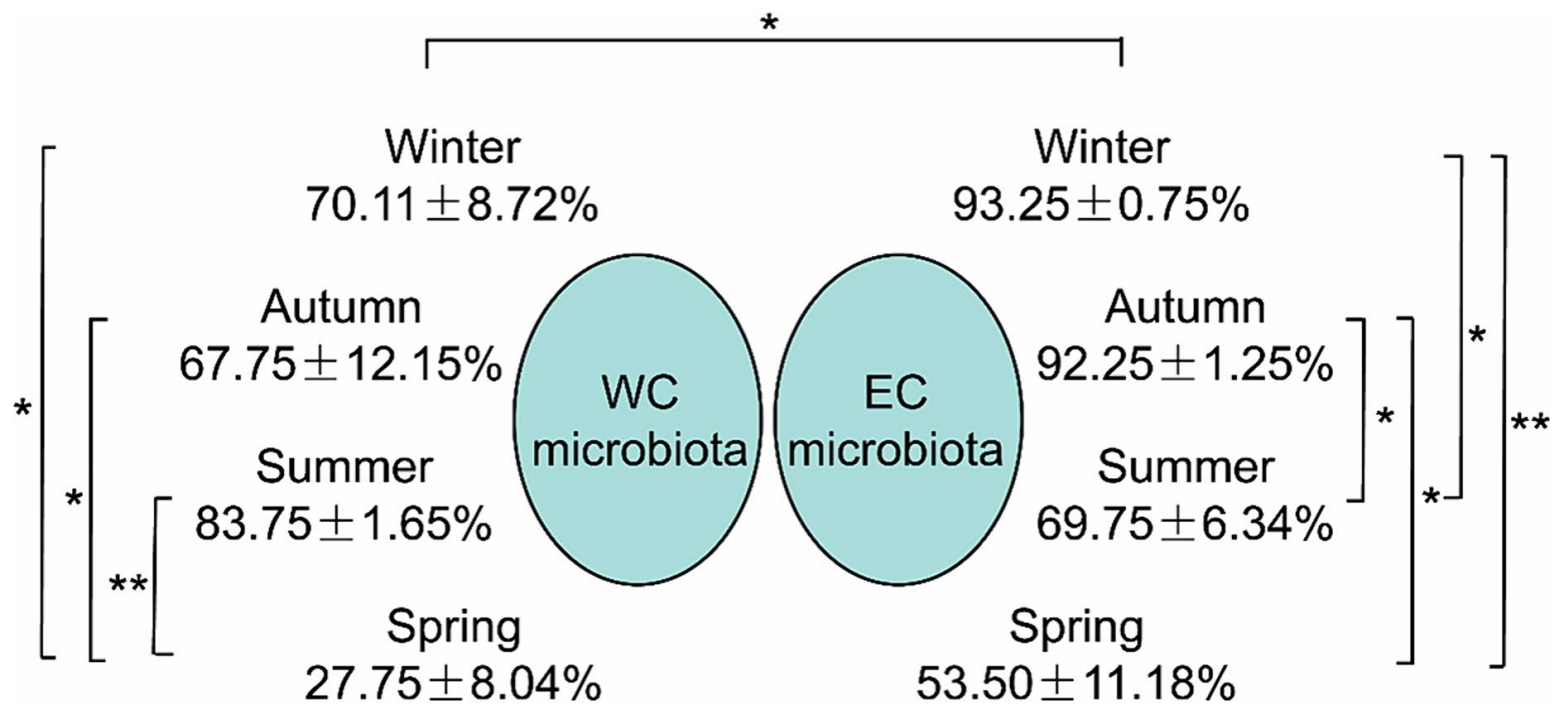
The proportion of planktonic eukaryotes in the microbiota from the rivers into the East Chaohu Lake and West Chaohu Lake detected using the SourceTricker. WC, West Chaohu Lake; EC, East Chaohu Lake. ^*^p < 0.05; ^**^p < 0.01.

SourceTracker analysis indicated that the proportion of PEs entering CL microbiota from the river microbiota was mainly affected by seasons, especially the lowest proportion in spring, and the highest proportion in summer in the WCL and in winter in the ECL (Figure 5).

**Figure 6:**
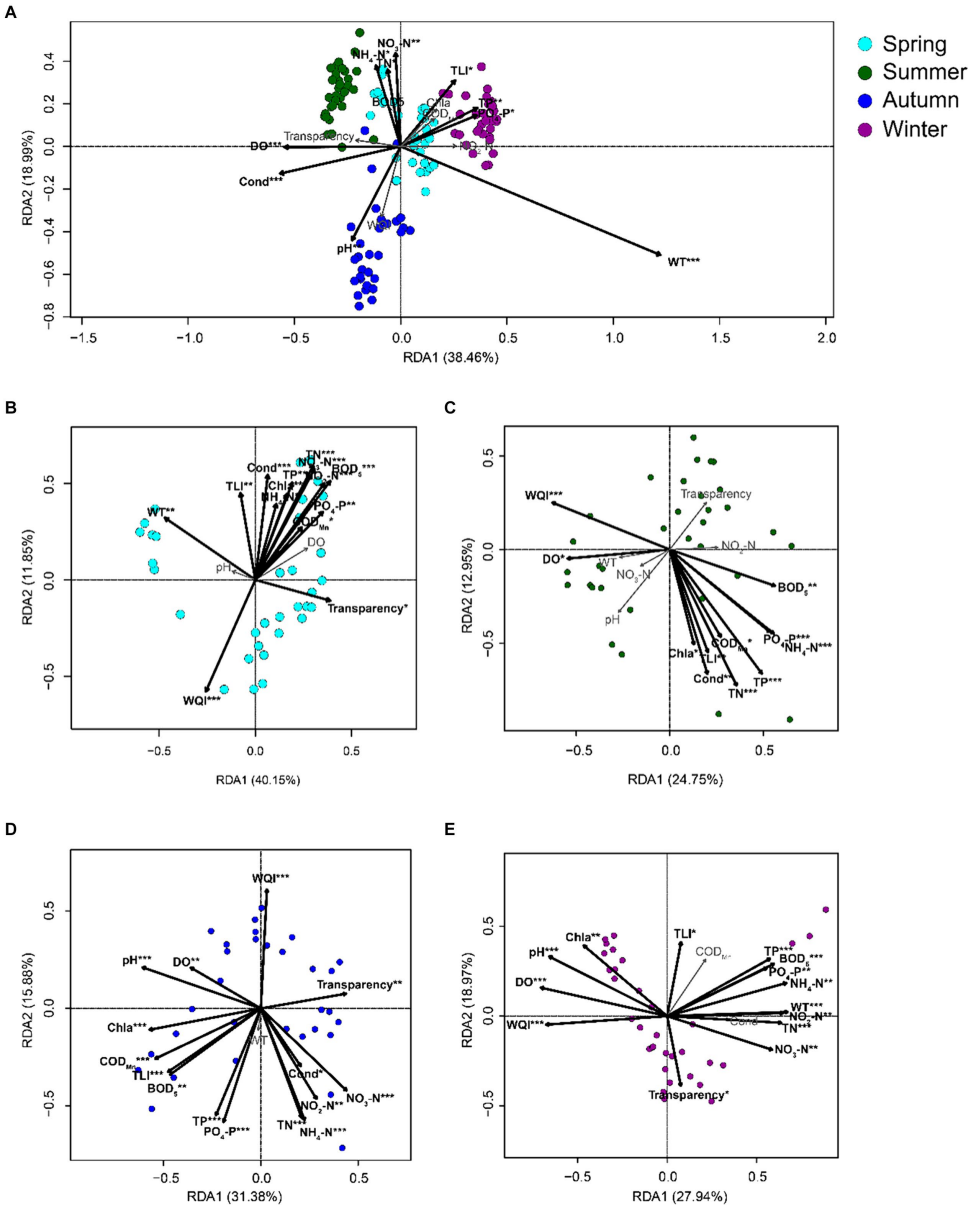
db-RDA profiles exhibit the correlations between freshwater environmental factors and the species in planktonic eukaryotic communities in the Chaohu Lake basin. **(A)** total samples; **(B)** spring samples; **(C)** summer samples; **(D)** autumn samples; **(E)** winter samples. TLI, trophic level index; WQI, water quality index; WT, water temperature; DO, dissolved oxygens; Cond, conductivity; TN, total nitrogen; TP, total phosphorus; COD_Mn_, permanganate index; BOD_5_, biochemical oxygen demand after five days; Chla, chlorophyll a. ^*^p < 0.05; ^**^p < 0.01; ^***^p < 0.001.

**Figure 7:**
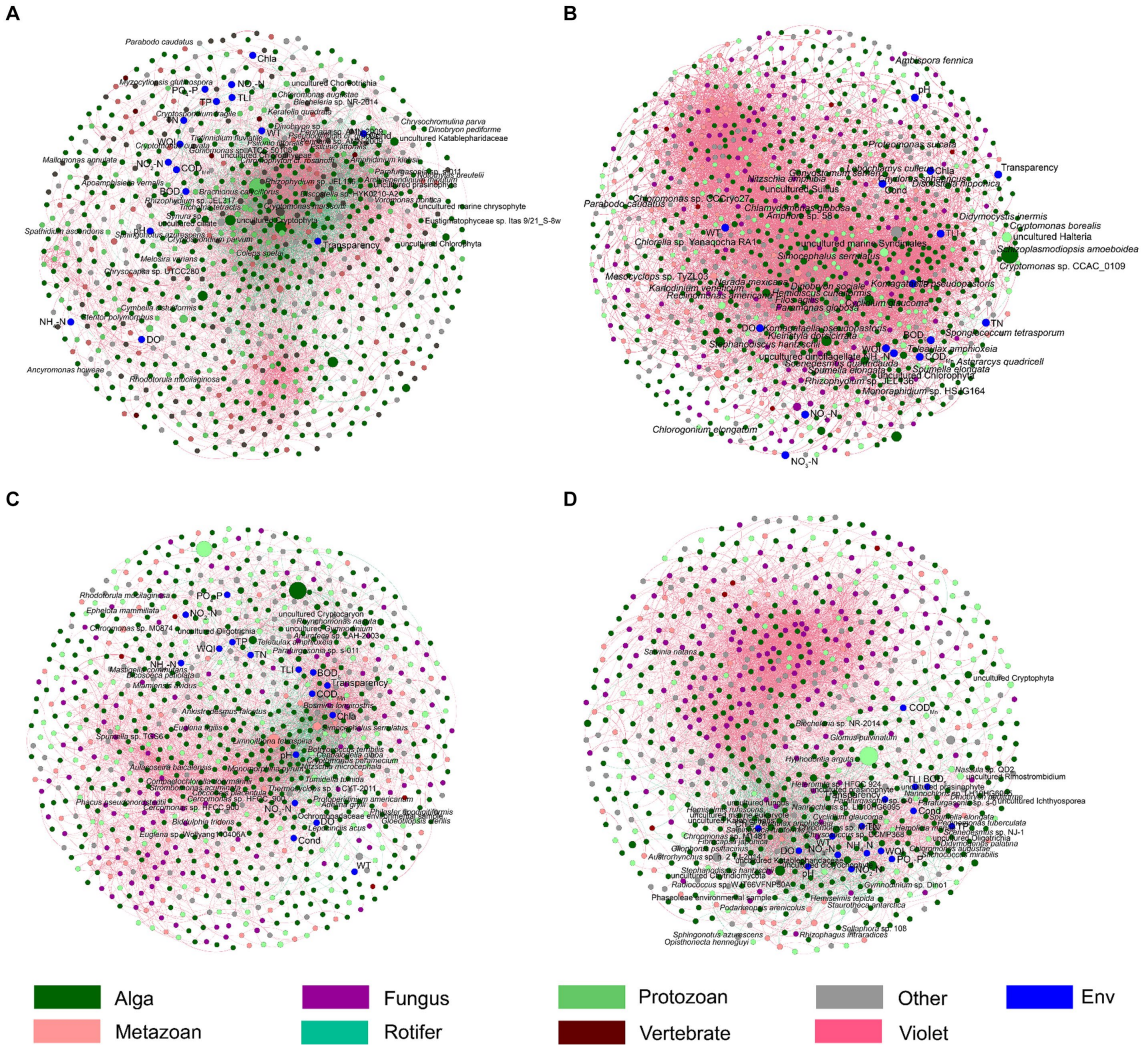
Co-occurrence networks shows the Spearman correlations between freshwater environmental factors and the dominant species in planktonic eukaryotic communities in the Chaohu Lake basin. **(A)** Spring; **(B)** Summer; **(C)** Autumn; **(D)** Winter. The red and green edges represent significantly positive and negative correlations, respectively. The diameters of the nodes represent their relative abundances.

The db-RDA results based on all samples of PECs and APPs indicated that WT, conductivity, DO, pH, PO_4_-P, TP, TLI, NO_3_-N, NH_4_-N, and TN significantly affected the PECs (*p* < 0.05; Figure 6A). The db-RDA results of the PECs and APPs in each season indicated that in spring, WQI, transparency, WT, BOD_5_, COD_Mn_, PO_4_-P, NO_2_-N, NO_3_-N, TN, TP, chl-a, NH_4_-N, conductivity, and TLI significantly affected the PECs (*p* < 0.05; Figures 6B, 7). In summer, WQI, DO, BOD_5_, PO_4_-P, NH_4_-N, COD_Mn_, TP, TN, conductivity, TLI, and chl-a significantly affected PECs (*p* < 0.05; Figures 6C, 7). In autumn, WQI, pH, DO, transparency, chl-a, COD_Mn_, TLI, BOD_5_, conductivity, NO_3_-N, NO_2_-N, TP, PO_4_-P, TN, and NH_4_-N significantly affected the PECs (*p* < 0.05; Figures 6D, 7). In winter, transparency, NO_3_-N, WQI, TN, NO_2_-N, WT, NH_4_-N, PO_4_-P, BOD_5_, TP, TLI, chl-a, pH, and DO significantly affected the PECs (*p* < 0.05; Figures 6E, 7). These results indicated that, although APPs had a significant impact on PECs, the impact of different APPs on PECs varied in different seasons.

**Figure 8:**
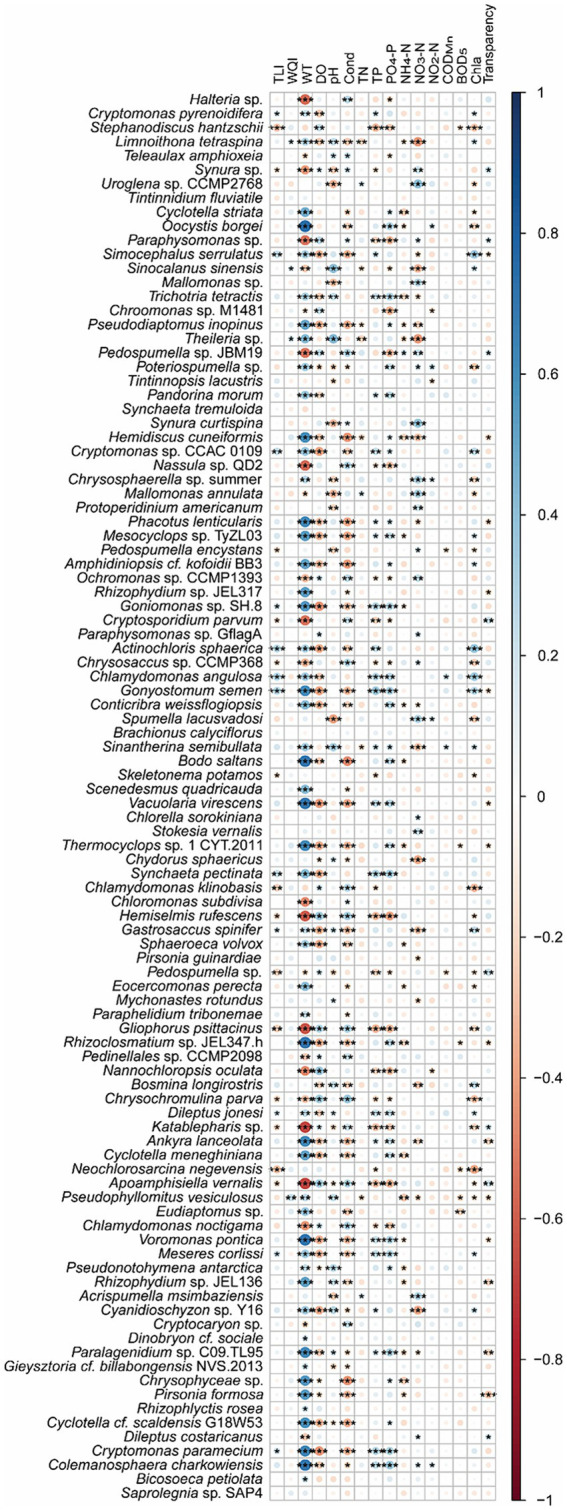
Spearman correlations between freshwater environmental factors and the top 100 species in planktonic eukaryotic communities in the Chaohu Lake basin. TLI, trophic level index; WQI, water quality index; WT, water temperature; DO, dissolved oxygens; Cond, conductivity; TN, total nitrogen; TP, total phosphorus; COD_Mn_, permanganate index; BOD_5_, biochemical oxygen demand after 5 days; Chla, chlorophyll a. ^*^p < 0.05; ^**^p < 0.01; ^***^p < 0.001.

Spearman correlation analysis and co-occurrence networks based on the Spearman correlation coefficients indicated that *Cryptomonas pyrenoidifera*, *Simocephalus serrulatus*, *Cryptomonas* sp. CCAC 0109, *Goniomonas* sp. SH-8, *Actinochloris sphaerica*, *Chlamydomonas angulosa*, *Gonyostomum semen*, *Synchaeta pectinata*, *Gastrosaccus spinifer*, *Dileptus jonesi*, *Meseres corlissi*, and *Cryptomonas paramecium* significantly positively correlated with TLI, and *Stephanodiscus hantzschii*, *Synura* sp., *Pedospumella encystans*, *Cryptosporidium parvum*, *Chrysosaccus* sp. CCMP368, *Skeletonema potamos*, *Chlamydomonas klinobasis*, *Hemiselmis rufescens*, *Pedospumella* sp., *Gliophorus psittacinus*, *Chrysochromulina parva*, *Katablepharis* sp., *Neochlorosarcina negevensis*, and *Apoamphisiella vernalis* significantly negatively correlated with the TLI (*p* < 0.05; Figure 8). However, only *Limnoithona tetraspina*, *Sinocalanus sinensis*, *Theileria* sp., and *Pseudophyllomitus vesiculosus* significantly positively correlated with the WQI (*p* < 0.05; Figure 8). *Uroglena* sp. CCMP2768, *Mallomonas annulata*, and *Acrispumella msimbaziensis* significantly positively correlated with TN and NO_3_-N, whereas *L. tetraspina*, *S. sinensis*, *Pseudodiaptomus inopinus*, *Theileria* sp., *Hemidiscus cuneiformis*, and *Sinantherina semibullata* significantly negatively correlated with TN and NO_3_-N (*p* < 0.05, Figure 8). Therefore, the increase in freshwater TN and NO_3_-N concentrations likely influenced these PEs. Similarly, *Trichotria tetractis*, *Cryptomonas* sp. CCAC 0109, *Phacotus lenticularis*, *Mesocvclops* sp. TvZL03, *Goniomonas* sp. SH-8, *C. angulosa*, *G. semen*, *S. semibullata*, *Vacuolaria virescens*, *S. pectinata*, *D. jonesi*, *Ankyra lanceolata*, *Voromonas pontica*, *M. corlissi*, *Paralagenidium* sp. C09.TL95, *C. paramecium*, and *Colemanosphaera charkowiensis* significantly positively correlated with TP and PO_4_-P, and *S. hantzschii*, *Paraphysomonas* sp., *Nassula* sp. QD2, *Ochromonas* sp. CCMP1393, *C. parvum*, *Chrysosaccus* sp. CCMP368, *H. rufescens*, *Pedospumella* sp., *G. psittacinus*, *Nannochloropsis oculata*, *C. parva*, *Katablepharis* sp., *A. vernalis*, and *Chlamydomonas noctigama* significantly negatively correlated with the TP and PO_4_-P levels (*p* < 0.05; Figure 8). However, because of the similar correlation between most bacteria and WT (Figure 8), the increase in freshwater TP and PO_4_-P concentrations probably only influenced the relative abundance of *S. hantzschii*.

Partial correlation analysis controlled WT showed that *S. hantzschii* (*p* = 0.002), *S. serrulatus* (*p* = 0.022), *Cryptomonas* sp. CCAC_0109 (*p* = 0.038), *P. encystans* (*p* = 0.010), *A. sphaerica* (*p* = 0.008), *C. angulosa* (*p* = 0.006), *G. semen* (*p* = 0.024), *S. potamos* (*p* = 0.020), *C. klinobasis* (*p* = 0.008), *Pedospumella* sp. (*p* = 0.018), and *N. negevensis* (*p* < 0.001) were significantly correlated with TLI, whereas *L. tetraspina* (*p* = 0.013), *Theileria* sp. (*p* = 0.002), and *P. vesiculosus* (*p* = 0.001) were significantly correlated with the WQI.

**Figure 9:**
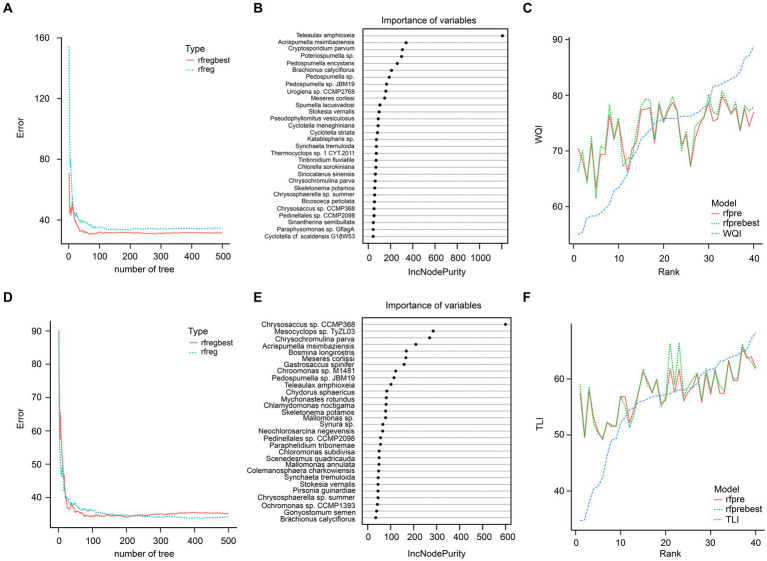
Random forest regression of the top 100 species in planktonic eukaryotic communities and WQI **(A–C)** and TLI **(D–F)**. **(A)** Prediction errors of WQI changed with number of tree; **(B)** the top 30 species greatest impacting on the predictive performance of WQI; **(C)** predictive results of WQI; **(D)** Prediction errors of TLI changed with number of tree; **(E)** the top 30 species greatest impacting on the predictive performance of TLI; **(F)** predictive results of TLI. rfreg and rfpre were the results of the random forest regression using R randomForest package with default parameters. Rfregbest and rfprebest were the results of the random forest regression using R randomForest package with optimized parameters obtained using tuneRF function.

Random forest regression results using the top 100 species showed that these species could not predict the WQI and TLI well, especially when the WQI and TLI were lower and higher, respectively (Figure 9).

## Discussion

The pollution of natural water has attracted much attention (Strokal et al., 2021; Zhang et al., 2021; Tang et al., 2022; Xie et al., 2022). Compared to rivers, lakes are more susceptible to eutrophication due to their low flow. Therefore, timely and effective identification of pollution sources is crucial for addressing lake pollution (Amiri et al., 2019; Cai et al., 2023). Our results indicated that the significant increases in TN, NH_4_-N, TP, and PO_4_-P levels in the NFR and PR probably were the primary cause of the higher nutrient content in the WCL compared to the ECL. However, although the concentrations of water NO_3_-N and NO_2_-N in the NFR and PR were significantly higher than those in other rivers, there was no significant difference in these nutrients between the WCL and ECL. These results implied that the treatment of TN and TP in the NFR and PR probably have effectively reduced their concentrations in the WCL.

Zooplankton play a crucial role in ecosystem dynamics (Bruce et al., 2006). As they are directly connected to HTLs, understanding how their distribution affects the entire ecosystem and their interactions with other planktonic groups can provide valuable insights into ecological models (Pinel-Alloul et al., 1988). For instance, Perissinotto and McQuaid (1992) found a link between the diurnal vertical migration of zooplankton and fish feeding habits. This migration also influences fish migration patterns and food availability (Lomartire et al., 2021). Additionally, zooplankton has a strong impact on the biomass stocks of other planktonic groups, as they can alter the concentration of prey (through consumption) and predator (through being consumed) populations, ultimately affecting fish biomass (Lomartire et al., 2021). Moreover, zooplankton are grazers for algae and bacteria, influencing their community. However, they also contribute to the nutrient recycling by providing phytoplankton with nitrogen and phosphorous (Lomartire et al., 2021). This nutrient recycling is essential for maintaining a healthy ecosystem. Furthermore, zooplankton plays a crucial role in the efficiency of the biological carbon pump, which regulates atmospheric carbon dioxide levels (Kwon et al., 2009). Our results indicated that season significantly influenced the PEC structures in the CL Basin, with the highest α-diversity observed in summer. Considering that microbiota diversity affects community stability, these results suggested that the structure and stability of PECs in rivers and lakes in the CL Basin varied throughout the seasons, potentially impacting their ecological functions. However, further verification is required to confirm these findings.

*Cryptomonas* is a widely distributed genus of mixotrophic freshwater microalgae found in lakes and ponds (Hornberger et al., 2023). It plays a crucial role in aquatic ecosystems as a grazer of bacterioplankton and zooplankton (Nishino et al., 2015). *C. angulosa*, a species within this genus, has been identified as a valuable biological resource and a potential tool for heavy metal bioremediation (Hwang et al., 2018). *Chlamydomonas*, a photosynthetic protist, is one of the simplest organisms in this group. It is capable of reproducing sexually or asexually and can grow through photoautotrophy, heterotrophy, or mixotrophy (Neupert et al., 2009). The abundance and occurrence of the freshwater raphidophyte *Gonyostomum semen* have been increasing, particularly in brown lakes, which are the most common type of lake in the boreal zone (Lebret et al., 2018). Our results indicated that these species were significantly positively correlated with TLI, indicating that the nutritional status of freshwater has a significant impact on algae and unicellular plankton in the PEC. Additionally, WQI has been shown to affect PEs at HTLs, such as *Limnoithona tetraspina* (Gould and Kimmerer, 2010), *Sinocalanus sinensis* (Islam et al., 2005), and *Pseudophyllomitus vesiculosus* (Shiratori et al., 2017).

Temperature plays a crucial role in the growth of *Chlamydomonas* cells, as they produce small heat shock proteins, chaperonins, and HSP70 heat shock proteins, and undergo other heat shock responses to cope with heat stress (Sekiguchi et al., 2018). Our results demonstrated a significant positive correlation between *C. angulosa* and WT (Figure 6), further supporting the understanding that temperature is a key factor in the growth of *Chlamydomonas* cells.

Certain species of zooplankton serve as bioindicators, meaning they are highly sensitive to disturbances in their natural environment (Lomartire et al., 2021). Due to their sensitivity, these organisms can quickly reflect disturbances, making them valuable indicators for various wetland areas worldwide (Serranito et al., 2016). For instance, species from the *Simocephalus* genus are commonly used as environmental indicators and standard test subjects in toxicological studies (Giesy et al., 1977), and can be found in a variety of habitats such as open littoral zones of ponds and lakes, semi-static tributaries of rivers and pools, and various types of puddles (Huang et al., 2014). However, our results from a random forest regression analysis showed that using the top 100 species did not accurately predict the WQI and TLI (Figure 7).

Although species composition is a crucial aspect of plankton ecology, it is equally important to consider changes in plankton biomass. This includes a comprehensive evaluation of the effects of human activities and climate change on plankton biomass. However, our study did not analyze biomass and it is recommended that future research prioritize this issue.

## Conclusion

The structure of PECs in the CL Basin was significantly influenced by the season, with the highest level of α-diversity observed during the summer. Although APPs had a significant impact on PECs, the impact of different APPs on PECs varied seasonally. The nutritional status of freshwater primarily affects the algae and unicellular plankton in freshwater PECs. However, the WQI affects PEs at HTLs. Using the top 100 species could not accurately predict the WQI and TLI through random forest regression. These results provide essential data for evaluating the impact of changes in WQI and TLI on the PEC in the CL Basin.

## Data availability statement

The datasets presented in this study can be found in online repositories. The names of the repository/repositories and accession number(s) can be found in the article/Supplementary material.

## Ethics statement

The manuscript presents research on animals that do not require ethical approval for their study.

## Author contributions

YZ: Funding acquisition, Writing – original draft, Writing – review & editing. MH: Data curation, Software, Validation, Writing – original draft. GD: Investigation, Software, Writing – original draft. KL: Investigation, Software, Writing – original draft. KH: Data curation, Writing – original draft. JZ: Investigation, Software, Writing – original draft. YL: Investigation, Software, Writing – original draft. YG: Data curation, Writing - review & editing. CZ: Data curation, Investigation, Writing – original draft. LW: Funding acquisition, Writing – review & editing, Writing – original draft.
