# Supplementary material for: Effects of season and water quality on community structure of planktonic eukaryotes in the Chaohu Lake Basin
Source: Front Microbiol. 2024 Aug 14;15:1424277. doi: 10.3389/fmicb.2024.1424277 (PMC11349697; doi:10.3389/fmicb.2024.1424277)
Supplement: Supplementary file 1 [file Data_Sheet_1.DOCX]

**Supplementary materials**

**Figure S1. Seasonal differences in water physical and chemical indicators.** * P<0.05; ** P<0.01; *** P<0.001.





**Figure S2. Differences in water physical and chemical indicators between rivers and Chaohu Lake.*** P<0.05; ** P<0.01; *** P<0.001.


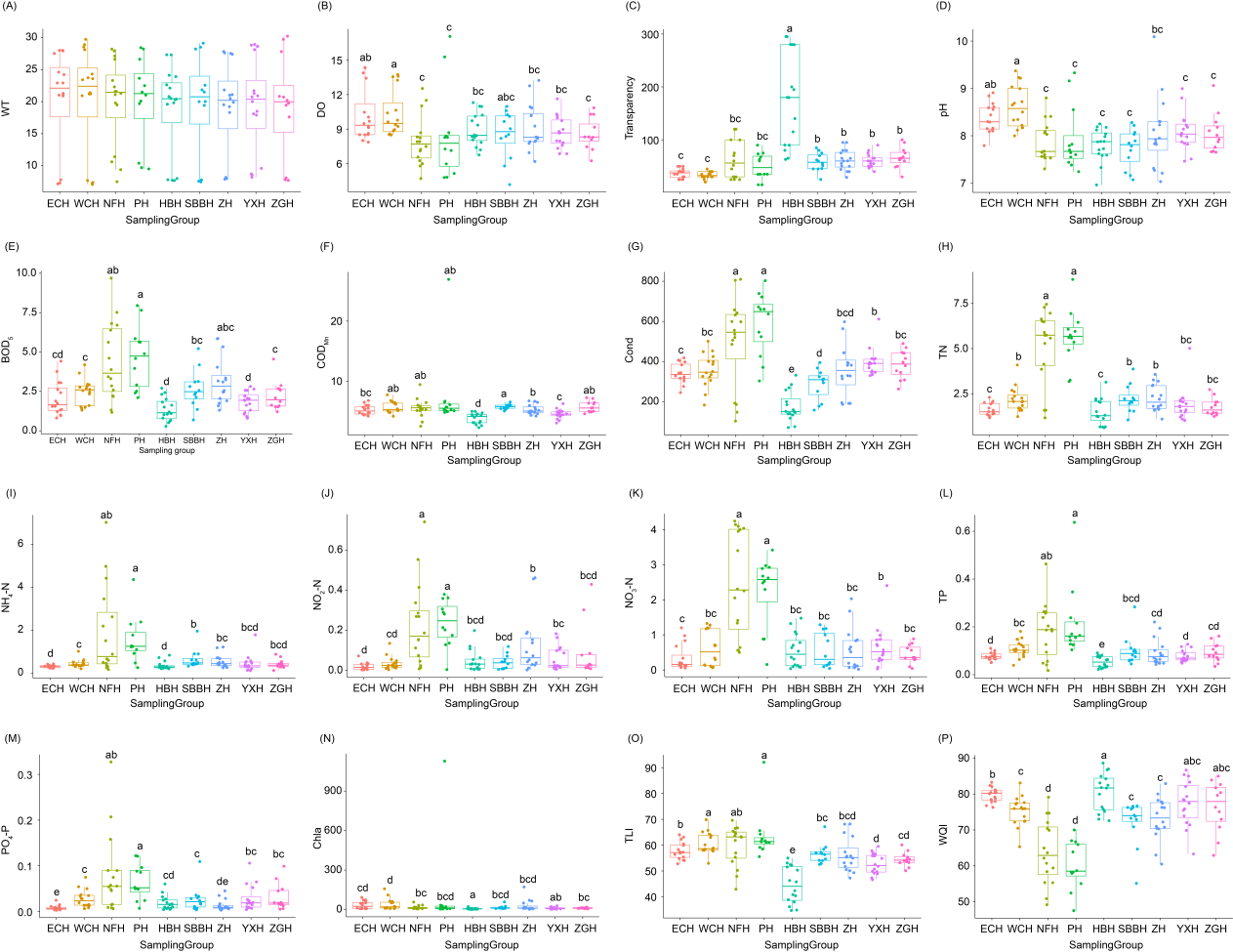


**Figure S3. Significant differences in the relative abundance of dominant phyla in planktonic eukaryotic communities in the Chaohu Lake basin.** (A) Annelida; (B) Apicomplexa; (C) Arthropoda; (D) Ascomycota; (E) Bacillariophyta; (F) Basidiomycota; (G) Blastocladiomycota; (H) Chlorophyta; (I) Chordata; (J) Chytridiomycota; (K) Cnidaria; (L) Ctenophora; (M) Euglenida; (N) Eustigmatophyceae; (O) Gastrotricha; (P) Mollusca; (Q) Mucoromycota; (R) Nematoda; (S) Nemertea; (T) Platyhelminthes; (U) Porifera; (V) Rotifera; (W) Streptophyta; (X) Xanthophyceae. * *P*<0.05; ***P*<0.01; ****P*<0.001.
